# Comprehensive genome characterization and expression analysis of the *DUF4228* gene family in Sorghum (*Sorghum bicolor* L.) under salt stress conditions

**DOI:** 10.7717/peerj.21175

**Published:** 2026-04-27

**Authors:** Esra Alther, Awadalla Abdalla Abdelmula, Hind Abdelmonim Elsanosi, Salma Mostafa, Guisheng Zhou

**Affiliations:** 1 University of Khartoum, Faculty of Agriculture, Shambat, Sudan; 2 Yangzhou University, The Joint International Research Laboratory of Agriculture and Agri-Product Safety, Ministry of Education of China, Jiangsu, China; 3 Alexandria University, Department of Floriculture and Landscape, Faculty of Agriculture, Alexandria, Egypt; 4 Yangzhou University, College of Horticulture and Landscape, Jiangsu, China

**Keywords:** Sorghum, Domains of unknown function (DUFs), Salinity stress, Expression analysis, Salt stress, Expression analysis

## Abstract

Sorghum (*Sorghum bicolor* L*.*) is recognized for its resilience to environmental adversities; the *DUF4228* gene family plays vital roles in development, growth, and response to stress in number of plant species such as * Arabidopsis thaliana,* and *Glycine max*, however, the systematic analysis of these gene families lacks reports in Sorghum. In this study, we identified and characterized members of the *DUF4228* gene family in the Sorghum genome through comprehensive bioinformatics analyses. A total of 25 * SbDUF4228* genes were discovered and mapped across eight chromosomes. Phylogenetic analysis divided these genes into six different groupings. Subcellular localization indicated that SbDUF4228 proteins are distributed across the nucleus, mitochondria, cytoplasm, and plasma membrane, indicating potential functional diversity. Expression analysis based on Fragments Per Kilobase of transcript per Million mapped reads (FPKM) values revealed that *SbDUF4228* genes are differentially expressed across distinct tissues. Furthermore, the promoter regions of the *DUF4228* gene were enriched with cis-regulatory elements associated with stress responses (MYB, MYC) and hormone signaling (ABA, MeJA). To further examine their role in abiotic stress response, we preformed quantitative real-time polymerase chain reaction (qRT-PCR) on seven *SbDUF4228* genes under salt stress conditions. The results indicated *SbDUF4228-4*, *SbDUF4228-14*, *SbDUF4228-1*, and *SbDUF4228-15* genes were significantly up-regulated following salt treatment, indicating their crucial role in salt stress tolerance. Overall, this study identifies key stress-responsive members of the *SbDUF4228* family and provides a foundation for future functional research and Sorghum improvement strategies.

## Introduction

Environmental disturbances are responsible for approximately 70% of annual crop yield losses (Shi, Wang & Liu, 2021), with abiotic stresses such as salinity being major contributors that disrupt plant growth and development (Mahalingam & Fedoroff, 2003; Sade et al., 2018). Although plants lack specialized sensory organs for directly perceiving environmental changes, they exhibit adaptive responses to cope with ecological stressors (Hamant & Haswell, 2017). To mitigate cellular damage caused by stress, plants activate complex defense mechanisms at molecular and cellular levels, which are essential for preserving physiological function under adverse conditions (Kim et al., 2014). The growing availability of genomic data has facilitated the identification and characterization of gene families susceptible to stress, encompassing FKBP, MAPK, NAC, PEPC, bHLH, Aux/IAA, bZIP, TIFY, HSF, and GRAS transcription factors (Zaynab, Khan & Li, 2021). Plant genomes encode several stress associated proteins with evolutionarily conserved domains that play crucial functions in diverse biological systems during stress (Zaynab, Khan & Li, 2021).

Domain of unknown function (DUF) proteins is characterized by the presence of the DUF domain and are widely found in plants (Bateman, Coggill & Finn, 2010; Luo et al., 2024). According to the Pfam database, which includes 18,259 protein entries, about 5,645 are classified as DUF families (Zhao et al., 2021). Notable DUFs include *DUF581* and *DUF724* in *Arabidopsis thaliana*, as well as *DUF1618*, *DUF936*, *DUF866*, *DUF810*, and *DUF221* in *Oryza sativa*. jointly termed DUF families (Su et al., 2021), account for approximately 24% (4,795 out of 19,632) of the protein families cataloged in the Pfam database (version 35.0) (Su et al., 2021). As of the 2021 update, functional roles have been identified for 1,132 DUF or UPF families, resulting in their reclassification and corresponding updates to Pfam annotations (Su et al., 2021). Members of the DUF family have emerged as important contributors to plant responses under abiotic stress conditions. For example, in rice (*Oryza sativa*), *OsDUF810.7* classified under *DUF2275* has been shown to enhance stress tolerance (Mistry et al., 2021), while other DUF containing proteins such as DUF966 (*OsDSR2*) and DUF1644 (*SIDP361*) are implicated in adaptive stress mechanisms (Luo et al., 2014). In *Arabidopsis thaliana*, *AT3g55990*, which encodes a DUF231 domain protein, acts as an inhibitor of cold adaptation by suppressing low-temperature adaptation responses (Li et al., 2016). Furthermore, RING-DUF1117 E3 ubiquitin ligases, encoded by *ARTDUF1* and *ARTDUF2*, exhibit reduced transcriptional activity under stress conditions (Xin et al., 2007). Similarly, *OsSIDP366* (*DUF1644*), a key regulator of salt and drought stress tolerance in rice, has been shown to confer increased resilience through modulation of regulatory pathways (Kim, Ryu & Kim, 2012). Notably, transgenic rice overexpressing *OsSIDP366* demonstrated improved tolerance to both salinity and drought stress (Luo et al., 2014). Additional DUF domains have been linked to other physiological roles; for instance, *DUF283* has been implicated in siRNA processing and RNA-directed DNA methylation (RdDM)-dependent gene silencing (Guo et al., 2016), while members of the *DUF538* family possess chlorophyll hydrolytic activity, potentially influencing photosynthetic efficiency (Dlakić, 2006). The overexpression of *TaSRHP*, a salt-inducible gene with a DUF581 domain, is linked to improved drought and salt stress tolerance in transgenic plants (Gholizadeh, 2016), further underscoring the functional versatility of DUF domain proteins in abiotic stress adaptation. To date, a total of 6,565 protein families has been annotated as DUFs, each identified by the DUF prefix followed by a unique numerical designation (*e.g.*, *DUF1*, *DUF2*) (Bateman, Coggill & Finn, 2010; Hou et al., 2013; Su et al., 2021). Among these, the DUF4228 family (Pfam accession: PF14009) has been extensively identified across various plant genomes (Schultz et al., 1998; Yang et al., 2020). Emerging evidence indicates that *DUF4228* genes are closely associated with responses to environmental stressors. In *Arabidopsis thaliana*, transcriptomic analyses under drought, salinity, low temperature, and osmotic pressure revealed that *AT1G10530*, *AT1G21010*, and *AT1G28190* exhibited notable differential expression, suggesting functional involvement in stress adaptation (Schultz et al., 1998). Moreover, ectopic overexpression of *GmDUF4228-70* in soybean (*Glycine max*) significantly improved seedling tolerance to drought and salinity, as supported by physiological indicators including altered proline content, levels of malondialdehyde (MDA), hydrogen peroxide (H_2_O_2_), and superoxide (O_2_^−^) accumulation (Yang et al., 2020). Conversely, the expression of *MsDUF* was significantly affected by NaCl, PEG6000, abscisic acid (ABA), and gibberellin (GA), which down-regulated its levels, suggesting a possible role as a negative regulator in abiotic stress responses (Leng et al., 2021). Additionally, recent findings indicate that DUF4228 family containing genes also contribute to plant resistance against fungal pathogens, further highlighting the diverse functional repertoire of this gene family (Wang et al., 2018).

Sorghum (*Sorghum bicolor* L.) is the fifth most significant cereal crop worldwide. It originated in Africa and is widely regarded as a model C4 plant species for genetic and physiological research demonstrates high photosynthetic efficiency and superior biomass productivity, enhancing its agronomic and ecological significance in agricultural systems (Didelon et al., 2020; Enyew et al., 2022; Mullet et al., 2014; Schmutz et al., 2010). It serves as an essential staple food source, especially in arid and semi-arid developing countries where drought and salinity frequently constrain agricultural productivity (Espitia-Hernández et al., 2022; Zhao et al., 2019). Compared to other major cereals, Sorghum demonstrates exceptional adaptability to a broad range of environmental conditions, enabling its cultivation in agroecological zones considered marginal for conventional agriculture (Tasie & Gebreyes, 2020). Its inherent salinity tolerance makes it an ideal system for studying plant responses to salt stress. Recent studies have revealed that flavonoid biosynthesis pathways are key contributors to salinity tolerance in Sorghum (Borrell et al., 2024). As a member of the Poaceae family, *Sorghum bicolor* exhibits notable resilience to multiple abiotic stressors, including salinity, heat, and drought (Huang, 2018; Ren et al., 2022). This adaptability facilitates its sustained cultivation under conditions that significantly hinder the growth and yield of other staple crops.

Among stress responsive gene families, the *DUF4228* gene family has garnered increasing attention due to its involvement in abiotic stress signaling and response mechanisms. Despite the potential importance of the DUF4228 domain, a systematic genomic analysis of this gene family in Sorghum has not been conducted. This knowledge gap hinders a comprehensive understanding of its contribution to Sorghum’s stress tolerance mechanisms. To address this, we performed a genome-wide identification and characterization of the *DUF4228* gene family in Sorghum, providing a foundational resource for future functional studies. The results offer new understanding of the functioning roles of *DUF4228* genes and establish a foundational resource for future research aimed at elucidating the chemical processes behind stress adaptation in Sorghum. Moreover, this work offers potential genetic targets for the development of stress resilient crop varieties *via* molecular breeding and biotechnological methodologies.

## Materials and Methods

### Genome identification of the *DUF4228* gene in Sorghum

To identify *DUF4228* gene family (PF14009) members in Sorghum, protein sequences of *DUF4228* genes from *Arabidopsis* were downloaded from the Phytozome database (https://phytozome.jgi.doe.gov/pz/portal.html, v13.1, accessed on 20 April 2025). These *Arabidopsis* proteins sequences DUF4228 were used as queries in a BLASTP search (http://blast.ncbi.nlm.nih.gov, 13.1, accessed on 21 April 2025) against the *Sorghum bicolor* BTx642 v1.1 as reference genome. In addition, DUF4228 related proteins were retrieved from the Phytozome database by keyword searching the Sorghum genome. The hidden Markov model (HMM) profile for the DUF4228 domain (PF14009) was retrieved from the Pfam database. This profile was used to query the Sorghum proteome (v3.1.1) using HMMER3.0. All resulting hits were verified for the presence of the complete DUF4228 domain using SMART (http://smart.embl-heidelberg.de) and PFAM (http://pfam.xfam.org/). Collectively, the both methods (Arabidopsis DUFs and PFAM domains) identified 25 members of the *DUF4228* family in the Sorghum genome.

To characterize the physicochemical properties of the identified SbDUF4228 proteins, computational analyses were conducted using the ExPASy ProtParam tool (https://www.expasy.org/resources/protparam, accessed on 25 April 2025) to determine key parameters, including amino acid content, theoretical isoelectric point (pI), and molecular weight (MW). Furthermore, subcellular localization predictions for the SbDUF4228 proteins were conducted utilizing the Plant-mPLoc service (http://www.csbio.sjtu.edu.cn/bioinf/plant-multi/, accessed on 27 April 2025), a platform specialized in predicting protein localization patterns in plant systems. These bioinformatics tools facilitated a comprehensive evaluation of the physicochemical attributes and intracellular distribution of the Sorghum *DUF4228* gene family members.

### Multiple sequence alignment and phylogenetic analysis

To facilitate phylogenetic classification and structural characterization of *SbDUF4228* genes, the protein sequences of 25 putative *SbDUF4228* members were retrieved from the Phytozome v13.1 genomic database (accessed on 28 April 2025). Then, a multiple alignment of sequences was executed by the MUSCLE algorithm implemented in MEGA version 11 (Tu et al., 2023). An unrooted phylogenetic tree was generated in MEGA version 11 utilizing the neighbor-joining (NJ) approach based on the p-distance model, incorporating 1,000 bootstrap replicates to assess branch support. The resultant tree was visualized and annotated utilizing the Interactive Tree of Life (iTOL) Platform. (https://itol.embl.de, accessed on 29 April 2025) (Tamura, Stecher & Kumar, 2021).

### Analysis (Ka/Ks) of *DUF4228* genes

The evolutionary divergence of paralogous *SbDUF4228* gene pairs was assessed by calculating pairwise non-synonymous (Ka) and synonymous (Ks) substitution rates. Protein sequences containing the DUF4228 domain were first retrieved from phytozome database (accessed 12 April 2025). Subsequently, the corresponding coding sequences were analyzed using the Ka/Ks Calculator 2.0 module within TBtools, applying the Nei-Gojobori (NG) method (accessed on 10 May 2025).

### Characterization of structural features, conserved motifs, and promoter Cis-Acting elements

To evaluate structural diversity and functional motifs within the *SbDUF4228* gene family, intron–exon structures and un-translated regions (UTRs) were analyzed using TBtools. Concurrently, Conserved protein motifs have been identified using MEME (https://meme-suite.org/meme/tools/meme, v5.3.3, accessed on 14 April 2025) (Zhou et al., 2023). The XML file containing motif pattern information, generated by MEME, was imported into TBtools to visualize the distribution of conserved motifs within the SbDUF4228 proteins (Bailey & Elkan, 1994).

The 1,500 bp upstream genomic DNA sequences of SbDUF4228 proteins were obtained from Phytozome v13.1. Subsequently analyzed using the PlantCARE database (http://bioinformatics.psb.ugent.be/webtools/plantcare/html/, accessed on 24 April 2025) to examine putative stress responsive and hormone related cis-regulatory elements in the promoter regions.

### Chromosomal mapping, duplication analysis, and synteny assessment

To determine the genomic distribution of *SbDUF4228* genes, their chromosomal locations were extracted from the Sorghum genome annotation file (GFF). Furthermore, duplication events of the Sorghum *SbDUF4228* gene were examined using TBtools software, and illustrated with Circos to depict the distribution and structure of *SbDUF4228* gene family members within the Sorghum genome (Chen et al., 2020a), with default parameters (E-value ≤ 1e−10, minimum of five gene pairs per syntenic block). Duplicate gene pairs were classified as whole-genome/segmental, tandem, proximal, or dispersed duplicates based on their genomic coordinates and collinearity. In addition, spatial visualization of their chromosomal localization was generated using TBtool. The Synteny evaluated was conducted utilizing the Multi Synteny Plot function in TBtools to construct comparative syntenic maps between *Sorghum bicolor* and two other species, *Arabidopsis thaliana* and *Glycine max.*

### Expression patterns of *SbDUF4228* proteins in sorghum tissues

The gene expression profiles of *SbDUF4228* genes were examined utilizing FPKM values obtained from the Phytozome database (accessed on 8 May 2025). The dataset encompassed five distinct Sorghum tissues: internode flag leaf, leaf boot stage, meristem at flag leaf, flowers, and seed boot stage. Gene expression visualization and clustering were performed using the heat map function in TBtools.

### Cultivation of plants and collection of tissue samples

The Sorghum cultivar (JinZa) was utilized for gene expression profiling. Seeds underwent surface sterilization and were germinated in Petri dishes, uniform seedlings were subsequently transferred to a hydroponic culture system supplemented with Hoagland nutrient solution (Hoagland & Arnon, 2018), which was refreshed every two days to ensure consistent nutrient provision. Both the seedling germination and the subsequent 72-hour salt treatment were conducted under controlled settings of 24 °C, 60% relative humidity, and a 16-hour light /8-hour dark photoperiod. Salt stress treatment (2 × Hoagland nutrient solution containing 200 mM NaCl) was initiated at the three-leaf developmental stage. Leaf samples were collected at 0 (control), 6, 12, 24, 48, and 72- hours post-treatment. Following each treatment, the leaves of nine plants from three separate biological replicates were harvested promptly flash-frozen in liquid nitrogen, and stored at −80 °C for future molecular analysis.

### RNA isolation and RT- PCR analysis

RNA was isolated from leaf tissue utilizing RNA Pure Plant Kit (DNase I) (Ca. # CW0559S, CWBIO, Taizhou, Jiangsu, China), following the manufacturer’s protocol to ensure genomic DNA removal. The quality of the RNA samples was assessed for degradation or contamination by 1% agarose gel electrophoresis. Additionally, the purity (A260/A280 ratio) and concentration of the RNA samples were determined using a Nanodrop ND-1000 spectrophotometer (V3.7.9). First-strand cDNA was generated from 1 µg of raw RNA utilizing the HiScriptIII RT SuperMix for qPCR** **(Cat. # R323-01, Vazyme, Nanjing, Jiangsu, China). Following the manufacturer’s instructions, reverse transcription reactions are performed at 50 °C for 15 min followed by 85 °C for 5 s. RT-qPCR primers were created based on the coding sequences of target genes *via* the Integrated DNA Technology site (https://sg.idtdna.com/pages/tools) and created by Qingke Biotechnology. The SbActin gene functioned as the internal reference for normalization (Table S1). Qualitative real-time polymerase chain reaction (qRT-PCR) was performed in triplicate using a CFX96 Real-Time PCR System (Bio-Rad, USA). Each 10 µl reaction contained 5 µl of 2x ChamQ SYBR qPCR Master Mix (Vazyme, Nanjing, China; Catalog # Q711), 1 µl of a 10 µM primer mix, 1 µl of cDNA, and 3 µl of RNase-free water. The thermal cycling protocol commenced with an initial denaturation at 95 °C for 30 s, followed by 40 cycles of denaturation at 95 °C for 10 s and annealing/extension at 60 °C for 30 s. A melting curve analysis was conducted immediately after amplification, with the temperature ramped from 65 °C to 95 °C at an increment rate of 0.5 °C/s.

### Data analysis

We used the Bio-Rad CFX Maestro software (version 4.1) to obtain the raw quantification cycle (Cq) values for each reaction Table S2. The relative expression levels of the target genes were quantified using the 2^−ΔΔCT^ method, with SbActin serving as the internal reference control. Statistical significance of expression differences was determined using a paired *t*-test (*p* < 0.05) on data from three independent biological replicates. Further details regarding the qPCR procedure are provided in the MIQE checklist (Table S3).

## Results

### Comprehensive characterization of *SbDUF4228s*

**Figure 1:**
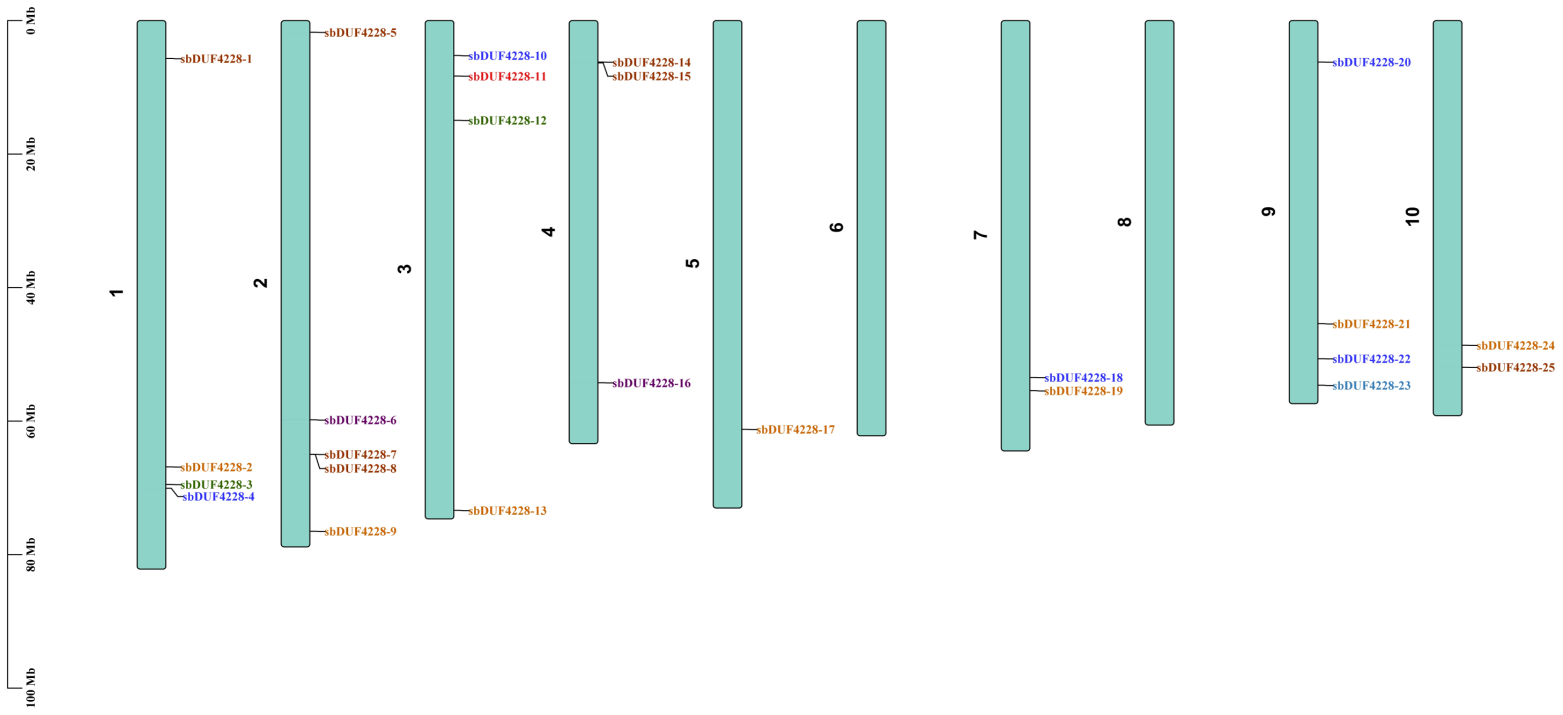
The chromosome numbers and names of the *SbDUF4228* genes, the scale on the left, in black, represent the length of each sorghum chromosome.

Through a comprehensive bioinformatics analysis, a total of 25 DUF4228 genes were discovered in the *Sorghum bicolor* BTx642 v1.1 genome and assigned those unique names (*SbDUF4228-1* to *SbDUF4228-25*) based on their chromosomal locations and relative linear arrangement, these genes were distributed unevenly across the chromosomes of Sorghum, along with all chromosomes contain 2–5 genes, except chromosome 5 contained only a single member, *SbDUF4228-1* (Fig. 1). The corresponding proteins exhibited substantial variation in sequence length, ranging from 112 to 294 amino acids. Their estimated molecular weights spanned from 11.2 to 29.4 kDa, while their theoretical isoelectric points (pI) varied between 4.88 and 11.22, indicating substantial diversity in physicochemical properties. Subcellular localization predictions revealed that SbDUF4228 proteins are distributed across various cellular compartments. Specifically, 12 proteins were predicted to localize predominantly in the nucleus, whereas four proteins were associated with both the plasma membrane and chloroplast. An additional four proteins were exclusively targeted to the chloroplast, and one protein was predicted to localize to the mitochondria. Interestingly, two proteins exhibited multi-compartment localization: one was detected in both the mitochondria and chloroplast, and another showed distribution across the mitochondria, cytoplasm, and chloroplast. These distinct localization patterns suggest functional divergence and specialization among *SbDUF4228* family members, potentially reflecting their involvement in different aspects of abiotic stress response and cellular regulation. Each SbDUF4228 protein was showed (Table S4).

### Sequence alignment and phylogenetic analysis of *SbDUF4228* gene family members

To investigate conserved features within the DUF4228 protein family, a multiple sequence alignment of representative homologs was performed using the MUSCLE algorithm, as implemented in MEGA version 11. The alignment identified several highly conserved regions across the sequences (Fig. S1). Specifically, cysteine and glycine residues were consistently preserved in the regions spanning positions 70–80 and 190–210, suggesting the presence of critical structural or functional motifs. Notably, conserved blocks were concentrated in the central and C-terminal regions (approximately residues 150–240 and 320–340), indicating that these domains may form the functional core of the DUF4228 family. In contrast, the N-terminal region exhibited considerable sequence variability, which could reflect differences in regulatory interactions or subcellular localization among homologs. The recurrent conservation of cysteine residues across the alignment suggests a potential role in disulfide bond formation or metal ion binding, both of which could contribute to the structural stability of the protein. These conserved patterns highlight key residues that may be critical for structural stability or biochemical function, warranting further investigation through structural modeling.

**Figure 2:**
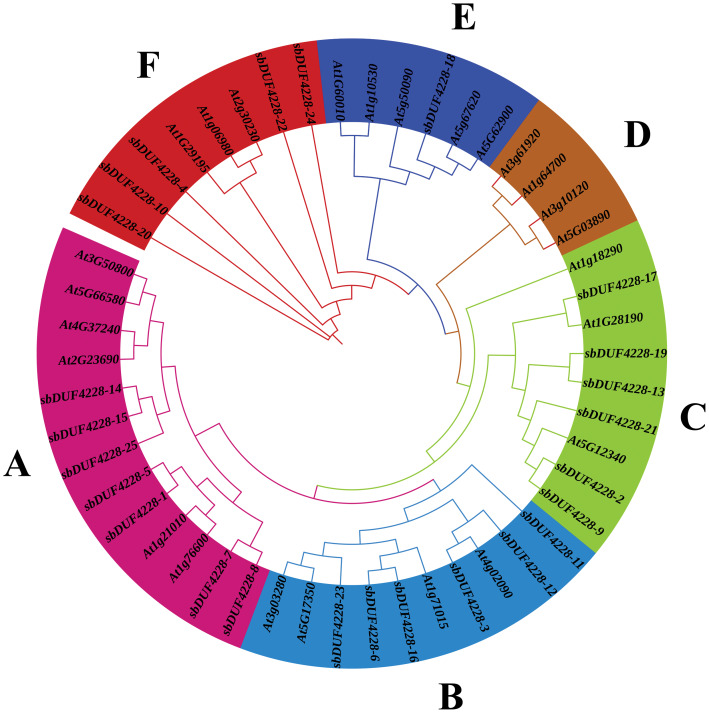
Phylogenetic tree of DUF4228 proteins from *Arabidopsis thaliana* and Sorghum bicolor was constructed using the Maximum Likelihood (ML) method. Distinct protein groups (A, B, C, D, E, and F) are highlighted by colored arcs.

The phylogenetic relationships between *SbDUF4228* and *DUF4228* homologs across plant species were examined using the Maximum Likelihood (ML) method, utilizing full length protein sequences. All *SbDUF4228* members were grouped into six distinct clusters, as showed in Fig. 2, with classification guided by branch lengths and tree topology. Intriguingly, a linear relationship was observed between groups and DUF4228 member abundance in Sorghum and its ancestral species (Fig. 2). This pattern implies that genomic duplication events may have driven the expansion and diversification of the *SbDUF4228* gene family.

### Gene structure and conserved elements of *SbDUF4228* family members

**Figure 3:**
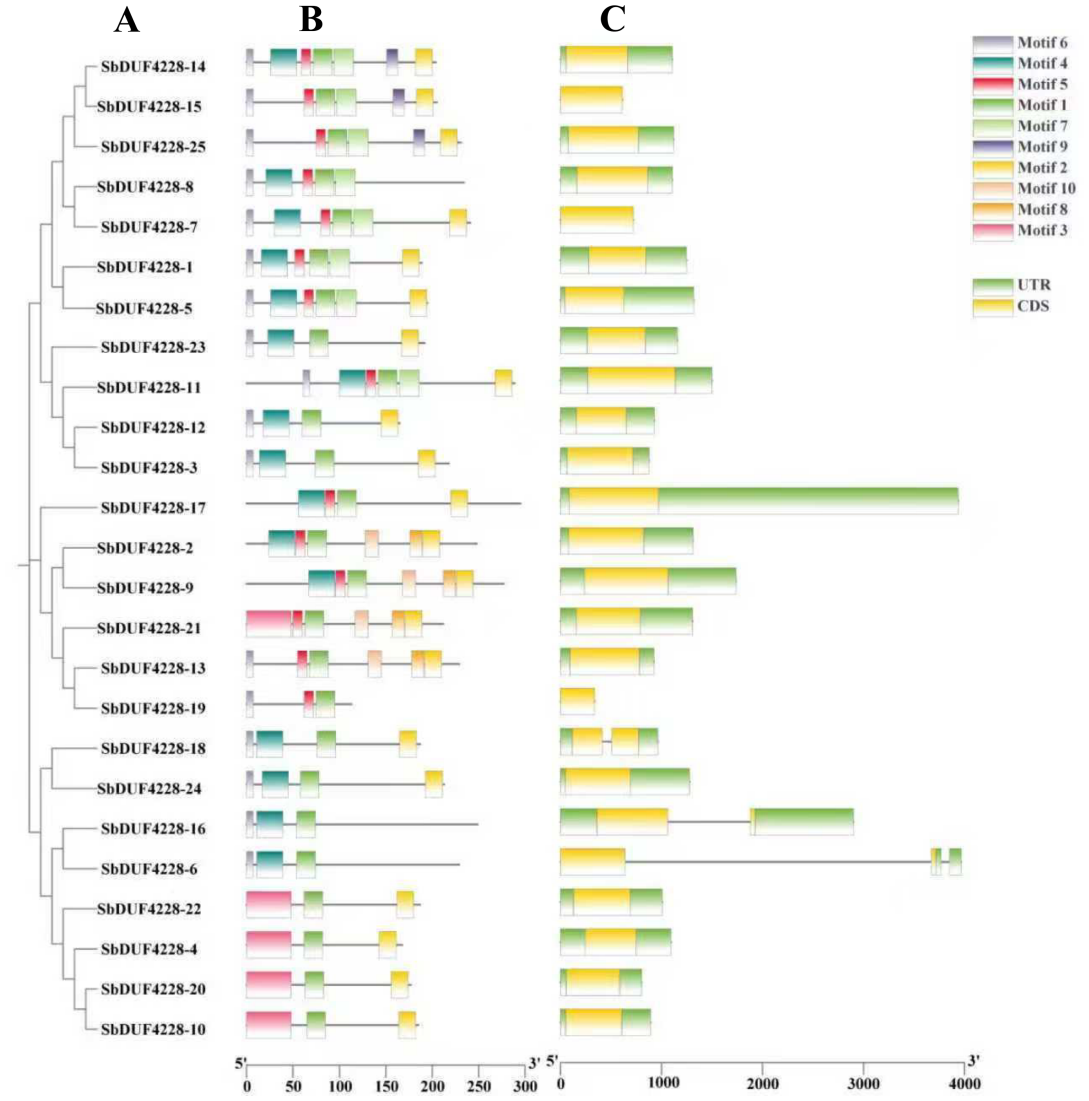
(A) Phylogenetic relationships, (B) conserved motif compositions, and (C) gene structures of *SbDUF4228* genes, distinct conserved motifs are illustrated as colored boxes. Green boxes denote untranslated regions (UTRs), while yellow boxes represent exons.

Conserved motifs within the SbDUF4228 protein family were predicted using the MEME online platform, revealing ten conserved motifs (Fig. 3B). Motifs 2, 4, and 6 were present in most family members, with Motif 1 present across all family members. In contrast, motifs 4–10 displayed a differential distribution exclusively presented in specific clusters. For example, Motif 3 was uniquely identified in Cluster 6, while Motif 9 was exclusive to Cluster 2, indicative of putative functional divergence among phylogenetic groups. Furthermore, exon-intron structural analysis, a key determinant of gene architecture and evolutionary dynamic, was conducted to elucidate diversification patterns within the gene family. Genomic annotation analysis of Sorghum using published GTF data revealed that *SbDUF4228* genes contain between 1 and 2 exons and up to 2 introns (Fig. 3C). While most genes feature both 3′- and 5′-UTRs, minorities retain UTRs at only one terminus or lack them entirely. Furthermore, genes within the same phylogenetic cluster exhibit conserved exon-intron architectures, implying that paralogs with shared conserved domains adopt analogous structural configurations (Fig. 3C).

### Homologous gene analysis of *SbDUF4228* genes and Ka/Ks analysis

**Figure 4:**
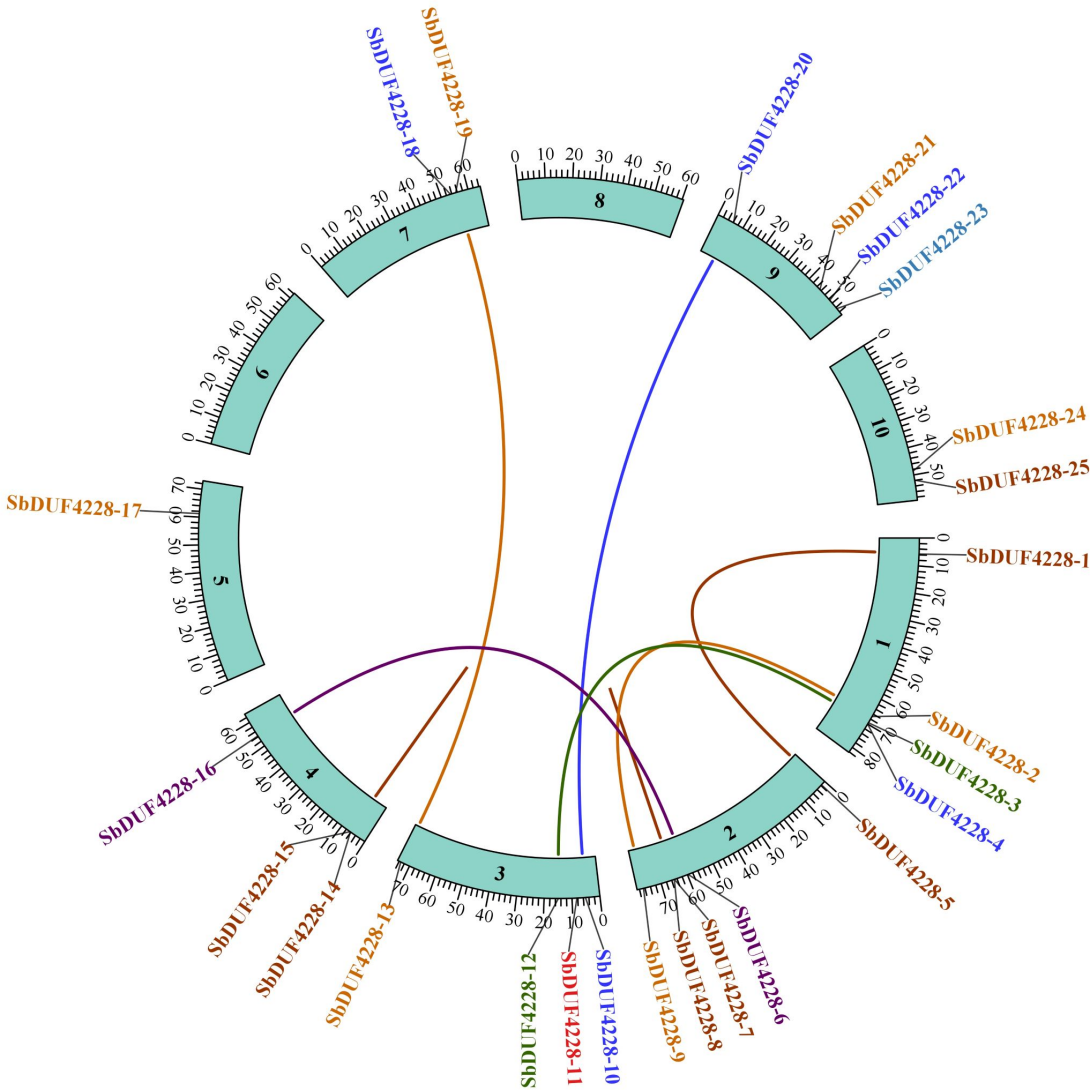
Homozygosity analysis of the sorghum *SbDUF4228* family. The different colors curve connecting the *SbDUF4228* genes indicates duplicate gene pairs in the sorghum *SbDUF4228* family.

**Figure 5:**
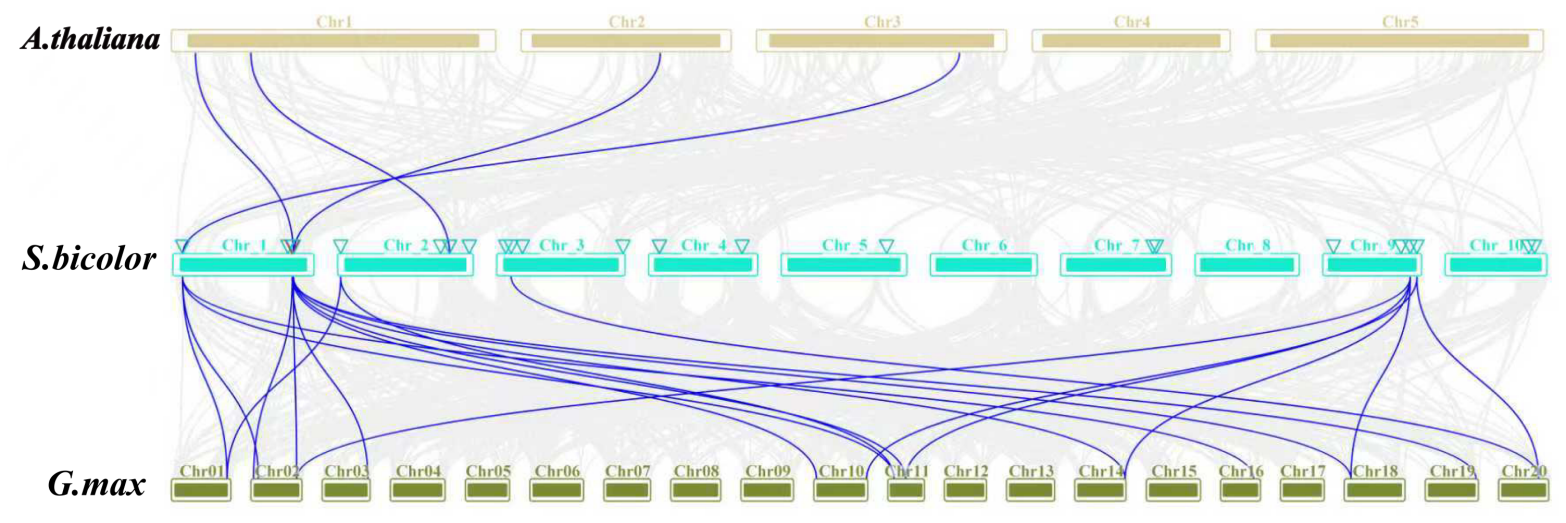
Synteny analysis of *DUF4228* genes among *Sorghum bicolor*, *Glycine max*, and *Arabidopsis thaliana*, gray lines in the background represent collinear blocks between S. bicolor and the other plant genomes, while blue lines highlight syntenic *DUF4228* gene pairs.

Homologous genes (HGs), defined as genes sharing structural and functional similarities due to common evolutionary ancestry, underscore the critical role of gene duplication in shaping functional divergence. Tandem duplication events were identified among 25 *SbDUF4228* paralogs (Fig. 4), suggesting that tandem duplication has substantially facilitated the proliferation of this gene family. To explore evolutionary conservation, a collinearity analysis was conducted to compare DUF4228 homologs across different plant species. This analysis revealed syntenic relationships and lineage-specific duplication patterns between *Sorghum bicolor, Arabidopsis thaliana,* and *Glycine max* (Fig. 5). To evaluate the evolutionary selection pressures acting on the *SbDUF4228* gene family, the proportion of nonsynonymous (Ka) to synonymous (Ks) substitution rates (Ka/Ks) was computed, an established metric for evaluating selective constraints on homologous genes. A Ka/Ks ratio below 1 reflects purifying selection, wherein deleterious mutations are eliminated to preserve gene function. A ratio equal to 1 reflects neutral evolution, suggesting the absence of selective pressure, while a ratio exceeding 1 implies positive selection, potentially associated with adaptive evolution. The analysis revealed that all *SbDUF4228* paralogous gene pairs displayed Ka/Ks ratios less than 1, signifying purifying selection, which may reflect functional conservation or specialization within the gene family (Table S5).

### Prediction of Cis-acting elements in *DUF4228* genes

To identify potential cis-acting elements of the *SbDUF4228* gene family we investigated the 1.5-kb upstream promoter regions using the PlantCARE database. Our analysis identified a total of 90 cis-regulatory elements, categorized into five functional groups: core promoter element regions (*e.g.*, TATA-box and CAAT-box), phytohormone-responsive elements (*e.g.*, abscisic acid, auxin, and gibberellin-related motifs), environment-responsive elements (*e.g.*, light, drought, and stress-related motifs), transcription factor-binding motifs, and tissue-specific regulatory elements (Table S6). These findings suggest that *SbDUF4228* genes may be regulated by a variety of developmental, hormonal, and environmental factors.

**Figure 6:**
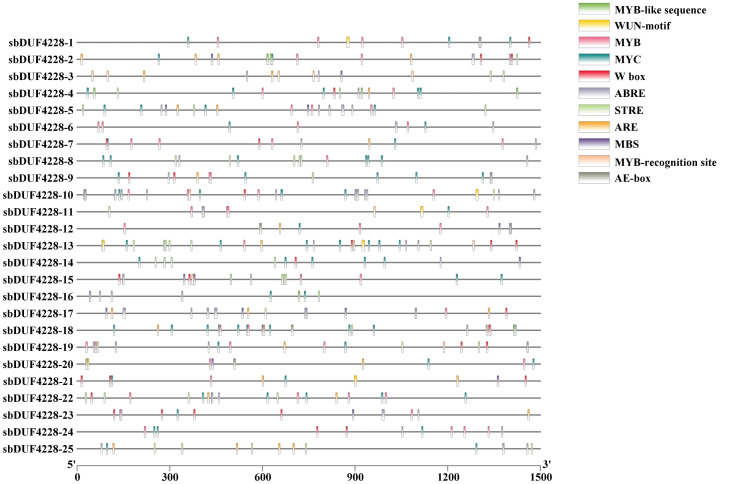
Analysis of cis-regulatory elements in *SbDUF4228* gene promoters Cis-acting regulatory elements were predicted within the 1,500 bp upstream promoter regions of *SbDUF4228* genes using the PlantCARE database. Distinct colors indicate elements grouped by functional categories, allowing for easy visual identification of motifs related to various biological processes.

The promoter regions of *SbDUF4228* genes exhibited a diverse array of cis-acting elements, indicating their potential involvement in multiple biological processes in Sorghum. Furthermore, stress-responsive motifs, such as MYC, MYB, Stress Response Element (STRE), and W-box (which are binding sites for WRKY transcription factors), were found to be highly enriched. To investigate the potential function of *SbDUF4228* genes in adaptation to salt stress, we focused on 11 cis-regulatory elements linked to environmental stress or stress-related phytohormonal signaling. These elements were consistently present across all *SbDUF4228* promoters (Fig. 6).

### Tissue-specific expression profiling of *SbDUF4228* genes

**Figure 7:**
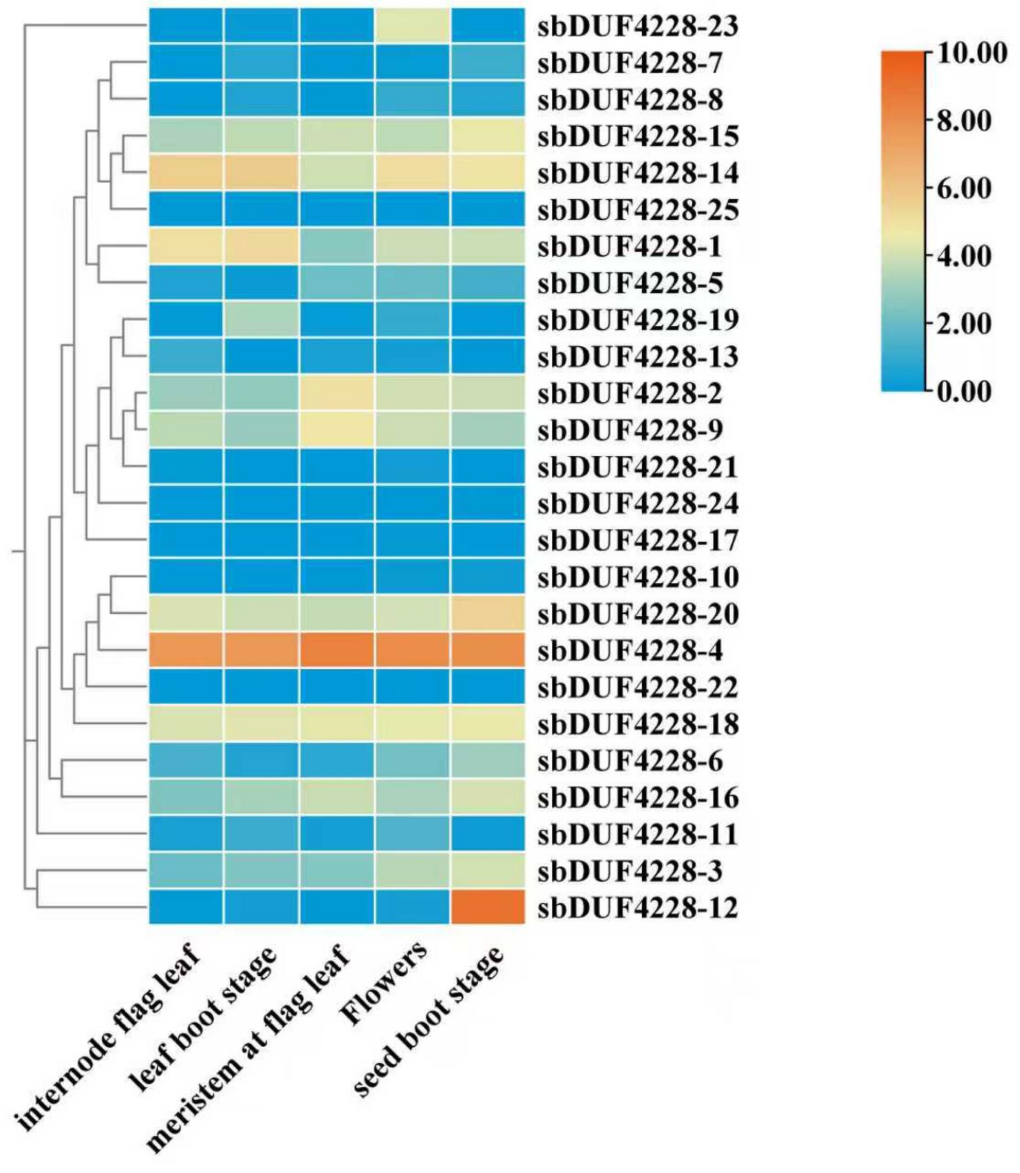
Analysis of expression patterns of *SbDUF4228* genes across five *Sorghum bicolor* tissues. The expression profiles based on FPKM (Fragments Per Kilobase of transcript per Million mapped reads) values. The color scale bars on the right indicate the expression levels of each gene, with the expression levels arranged horizontally.

To examine the expression profiles of *SbDUF4228* genes, expression data (FPKM values) were obtained from the Phytozome database. The analysis included five distinct Sorghum tissues: internode flag leaf, leaf boot stage, meristem at flag leaf, flowers, and seed boot stage (Fig. 7 and Table S7). The majority of *SbDUF4228* genes were expressed in at least one tissue. However, certain genes, such as *SbDUF4228-10*, *SbDUF4228-21*, *SbDUF4228-22*, *SbDUF4228-24*, and *SbDUF4228-17*, exhibited minimal or no expression across all tissues examined. In contrast, genes including *SbDUF4228-1*, *SbDUF4228-2*, *SbDUF4228-3*, *SbDUF4228-9*, *SbDUF4228-14*, *SbDUF4228-15*, *SbDUF4228-16*, *SbDUF4228-18*, and *SbDUF4228-20* demonstrated widespread expression across all tissues. Furthermore, *SbDUF4228-4* exhibited high expression levels in all tissues analyzed, while *SbDUF4228-12* was up-regulated in seed boot stage, as showed in Fig. 7. These results highlight the differential expression of *SbDUF4228* genes in diverse tissues, suggesting functional diversification and tissue specific regulatory roles.

### Evaluation of *SbDUF4228* gene expression in response to salt stress

**Figure 8:**
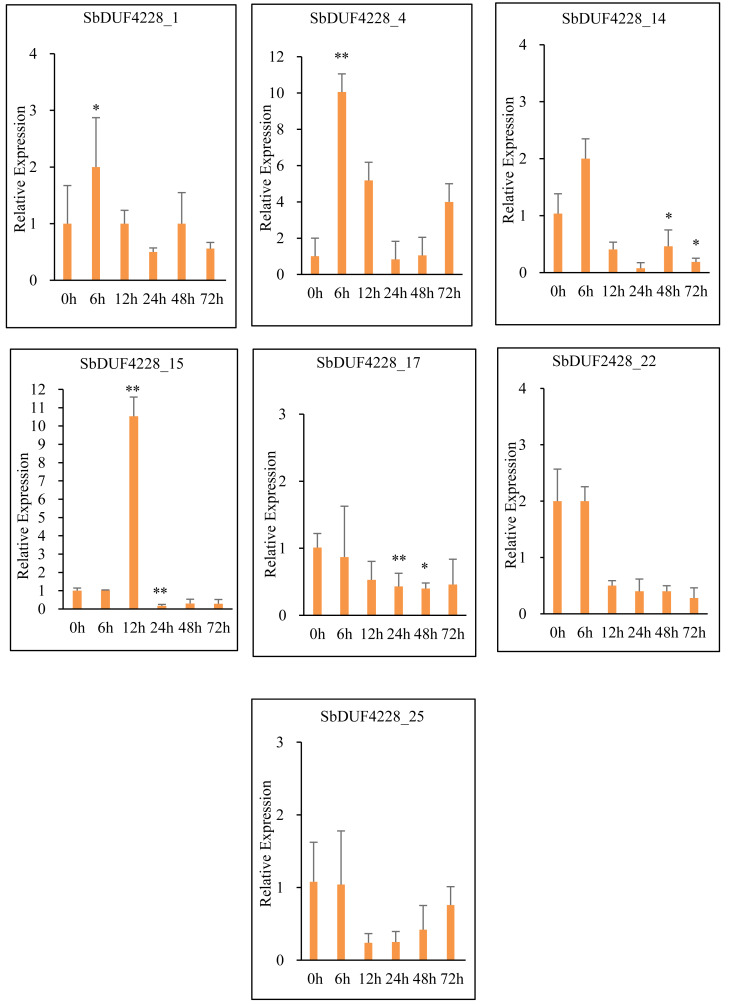
Expression profiles of seven *SbDUF4228* genes under 200 mM NaCl level, at 0, 6, 12, 24, 48, and 72 h after treatments measured by qRT-PCR, Expression was normalized to SbPP2A. (Actin). Error bars show Standard Deviation; asterisks indicate significant differences *vs.* 0 h by t-test (*p < 0.05; **p < 0.01).

**Figure 9:**
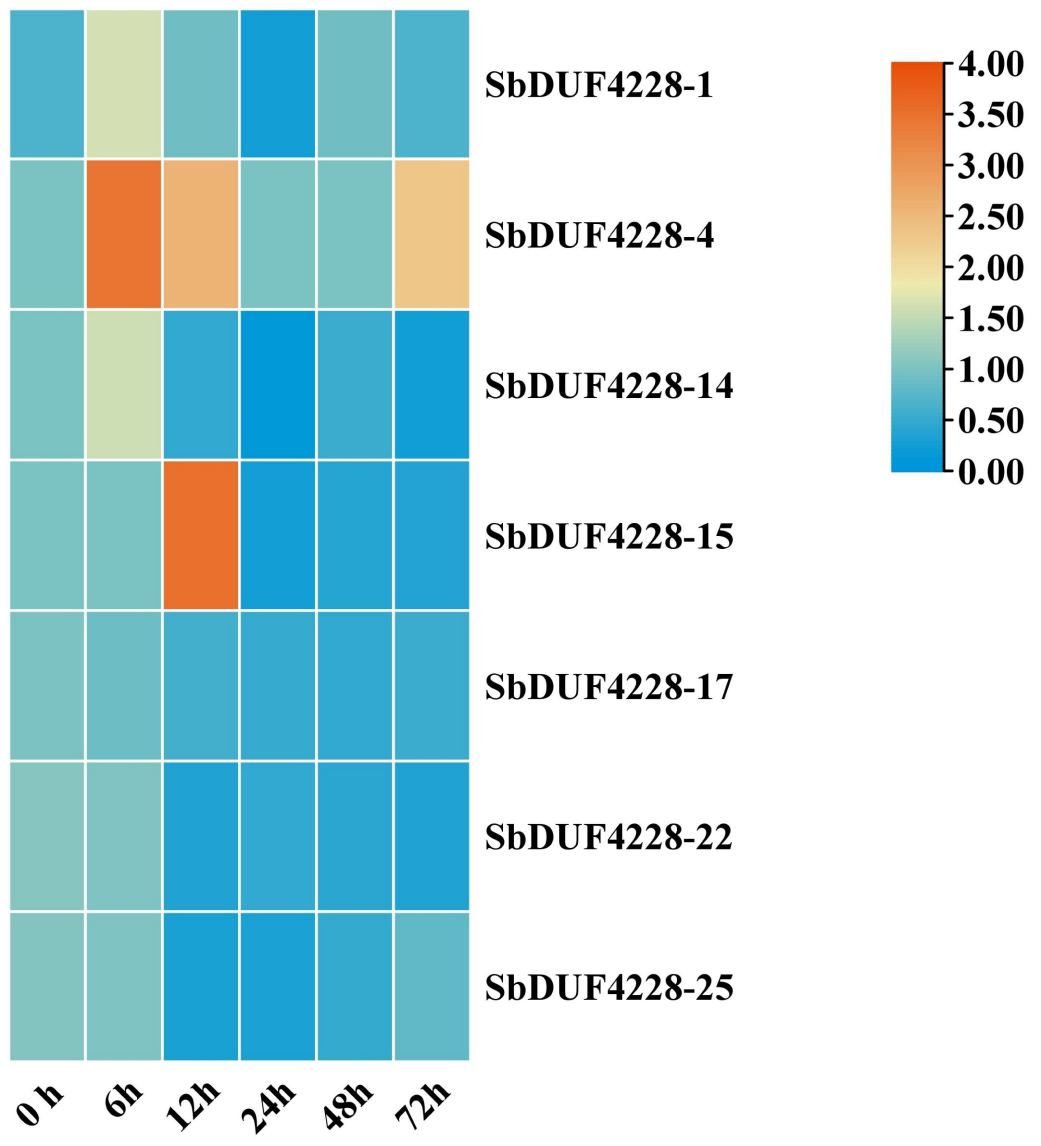
Heatmap visualization was performed using log-transformed mean expression values derived from three independent qRT-PCR replicates. The color gradient represents the relative signal intensity of each gene across treatments.

To investigate the potential role of DUF4228-domain in the salt stress response, we analyzed the expression of seven selected *SbDUF4228* genes *via* qRT-PCR (Figs. 8 and 9). Exposure to high salinity (200 mM NaCl) elicited distinct differential expression patterns among the selected genes, three genes exhibited altered expression. Specifically, *SbDUF4228-1*, *SbDUF4228-4*, and *SbDUF4228-14* were significantly up-regulated at the 6-hour time point compared to the control (0 h), suggesting their involvement in the early stress response. The expression of *SbDUF4228-15* significantly up-regulated at 12 h (Figs. 8 and 9). In contrast, *SbDUF4228-17*, *SbDUF4228-22*, and *SbDUF4228-25* were significantly down-regulated throughout the stress period, suggesting that their suppression may be necessary for physiological adaptation to salinity.

## Discussion

The Domain of Unknown Function (DUF) protein families comprise a group of proteins whose functions remain largely uncharacterized but are increasingly recognized for their potential roles in the development of plants and reactions to abiotic stress. The *DUF4228* gene family, recently identified in higher plants, has attracted attention due to its potential involvement in plant growth, development, and abiotic stress resilience (Zheng, Dang & Sui, 2023).

Sorghum *(Sorghum bicolor* L*.)* a cereal crop native to Africa, ranks as the fifth most important grain crop worldwide. It is widely recognized for its exceptional tolerance various abiotic stresses, including drought, floods, salt stress, soil degradation, and high temperatures (Bakari et al., 2023).

To date, a genome-wide identification and characterization of the *DUF4228* gene family in Sorghum has not been reported. Therefore, in this research, we conducted a systematic comprehensive genome characterization of the *DUF4228* gene family in *Sorghum* using bioinformatics approaches. 25 *SbDUF4228* genes were recognized from the Sorghum reference genome. Comparative analysis revealed the presence of 25 *DUF4228* genes in *Arabidopsis thaliana*, 81 in *Glycine max*, and 308 *DUF4228* genes in four species of Gossypium, indicating significant variation in gene family size across species (Leng et al., 2021; Yang et al., 2020; Zheng, Dang & Sui, 2023). Phylogenetic analysis, involving *S. bicolor*, *A. thaliana*, and *G. max*, allowed for the classification of these genes into distinct evolutionary groups, providing insights into their potential functional divergence.

The variety in motifs and their numbers in the *SbDUF4228* gene family may contribute to the functional diversity of these genes. The present of motif 1 in all DUF4228 proteins, suggesting it is crucial for the identification of the Sorghum *DUF4228* genes. Analysis of gene structure revealed that *SbDUF4228* genes contain between one and two exons and up to two introns. This exon/intron structure differs from that observed in *Glycine max*, where *GmDUF4228-70* was found to contain seven introns (Yang et al., 2020).

Homologous genes (HGs) characterized by analogous structures and biological functions across various species or within the same species due to a shared evolutionary ancestry, are crucial in elucidating gene function. To explore the homology of the *SbDUF4228* genes with dicotyledonous plants (*G. max* and *A. thaliana*) we performed covariance analyzed, our results revealed that *SbDUF4228* genes share four homologous pairs with *Arabidopsis thaliana* and twenty homologous pairs with *Glycine max*. This substantial numerical disparity indicates a significant expansion of the DUF4228 family in the soybean genome relative to that of *Arabidopsis*.

Gene duplication, in particular, is a key factor in shaping gene function. In this study, synteny analysis between the Sorghum genome and two other plant genomes revealed that all *DUF4228* duplicated genes have experienced purifying selection throughout the duplication process; this indicates that the role of these duplicated *DUF4228* genes has likely remained conserved through subsequent evolutionary events. These findings align with previous studies, such as those on potato (*Solanum tuberosum*) *StDUF4228* genes, where gene duplication also exhibited both positive and purifying selection pressures (Zaynab, Khan & Li, 2021).

Cis-acting components within promoter regions of genes play a crucial role in the transcriptional regulation of gene expression by interacting with transcription factors. These elements are involved in regulating gene expression during various physiological mechanisms, such as growth, development, and responses to environmental stress. Research indicates that members of the *DUF4228* gene family are crucial for plant growth, development, and stress responses (Zheng, Dang & Sui, 2023). In this study, we examined the cis-acting elements within the 1,500-bp upstream promoter region of *Sorghum bicolor DUF4228* genes. Our analysis revealed 90 cis-acting elements can be classified into five functional groups, such as light response, biotic/abiotic stress, growth and development, particular region, and plant hormone response. This indicates that the *SbDUF4228* genes likely play a significant role in plant growth, stress responses, and development. A similar finding was reported in Gossypium *DUF4228* genes, where cis-acting elements related to growth and development, specific regions, and plant hormone responses accounted for the most substantial proportion of the identified elements (Leng et al., 2021). Methyl jasmonate (MeJA) and abscisic acid (ABA) are essential phytohormones that significantly influence several signaling pathways, governing both physiological and molecular processes in plants. These hormones are pivotal to plant defense systems against biotic challenges and also aid in alleviating abiotic conditions, including drought, salinity, vernalization, and heavy metal exposure (Chen et al., 2020b; Rehman et al., 2023). In this research, we identified a significant presence of cis-acting elements associated with methyl jasmonate (MeJA) and abscisic acid (ABA) in the promoter regions of the *SbDUF4228* gene family. This result is consistent with previous analyses of the Gossypium *GhDUF4228* promoter (Lv et al., 2022). Furthermore, additional elements identified in the *SbDUF4228* gene promoters are linked to abiotic stress responses; this result aligns with the findings of prior investigations into responses to dehydration, salt stress, and ABA signaling (Wang et al., 2019).

Numerous studies have demonstrated that members of the DUF family are linked to plant stress resistance (Yang et al., 2020; Zheng, Dang & Sui, 2023). The overexpression of the *GmDUF4228-70* gene enhanced the expression of marker genes in response to both drought and salt stressors (Yang et al., 2020). In Gossypium, the *GhDUF4228-67* gene has been shown to be a positive regulator of cotton reaction to salt stress (Lv et al., 2022). Addition, in *A. thaliana*, the *ATDUF4228* genes may be implicated in the processes of plant tolerance to abiotic stressors (Yang et al., 2020). To further investigate the response of the *SbDUF4228* gene family to salt stress, qRT-PCR an analysis was performed on seven selected *SbDUF4228* genes. The results showed that under high salinity conditions (200 mM NaCl), most of the selected *SbDUF4228* genes were initially up-regulated, and followed by a gradual decrease in expression, suggesting their potential role in the early response to salt stress. Notably, *SbDUF4228-1*, *SbDUF4228-4*, *SbDUF4228-14*, and *SbDUF4228-15* exhibited significant up-regulation, with the greatest decline observed at 48 h. The expression of *SbDUF4228-15* showed an up-regulation and significantly higher than that of 0 h (control) at 12 h. In contrast, *SbDUF4228-17*, *SbDUF4228-22*, and *SbDUF4228-25* were significantly down-regulated compared to the control (0 h). These results suggest that the *SbDUF4228* gene family exhibits distinct, temporal, and stress specific expression patterns in response to salinity. The identification of *DUF4228* genes in the *Sorghum bicolor* genome, with their differential responses to salt stress, underscores the potential role of this gene family in salinity adaptation. Collectively, these findings provide valuable insights into the functional significance of *SbDUF4228* genes and may inform strategies for developing Sorghum varieties with enhanced tolerance to salinity.

## Conclusions

DUF4228 proteins are crucial for plant responses to abiotic stress. In this study, a total of 25 *SbDUF4228* genes in *S. bicolor* were found *via* bioinformatics research and classified into six categories (Group A, B, C, D, E, and Group F). Predictions of subcellular localization revealed that these genes are primarily situated in the nucleus, plasma membrane, chloroplast, mitochondria, and cytoplasm. The examination of gene architecture, chromosomal localization, and phylogenetic connections demonstrated that the *SbDUF4228* gene family is significantly preserved across the evolutionary history of Sorghum. Promoter analysis revealed many cis-acting factors linked to growth, development, environmental stress responses, and control of hormones. Gene expression pattern analysis suggested that the *SbDUF4228* gene family plays a regulatory role in the growth and development of Sorghum. qRT-PCR further validated that the expression of *SbDUF4228* genes is both temporally dependent and specific to salt stress. The expression profiles of *SbDUF4228* genes indicated that *SbDUF4228-1*, *SbDUF4228-4*, *SbDUF4228-14*, and *SbDUF4228-15* are particularly important in reaction to salinity stress, suggesting their potential role in Sorghum adaptation to environmental stresses.

##  Supplemental Information

10.7717/peerj.21175/supp-1 Supplemental Information 1 Description of primers utilized for the Real time PCR

10.7717/peerj.21175/supp-2 Supplemental Information 2 Raw data

10.7717/peerj.21175/supp-3 Supplemental Information 3 Checklist

10.7717/peerj.21175/supp-4 Supplemental Information 4 Physicochemical properties and subcellular localization of predicted Sorghum bicolor DUF4228 proteins

10.7717/peerj.21175/supp-5 Supplemental Information 5 Ka/Ks Analysis in DUF4228 Protein

10.7717/peerj.21175/supp-6 Supplemental Information 6 Details of the cis-elements identified in DUF4228 gene family

10.7717/peerj.21175/supp-7 Supplemental Information 7 The SbDUF4228 gene expression profile in variant tissue of sorghum

10.7717/peerj.21175/supp-8 Supplemental Information 8 Protein sequence alignment of the 25 SbDUF4228 homologs in sorghum

## Abbreviations

DUF: Domain of unknown function
HMM: Hidden Markov model
SbDUF4228: *Sorghum bicolor* DUF4228
AtDUF4228: *Arabidopsis thaliana* DUF4228
pI: Isoelectric point
MeJA: Methyl Jasmonate
ABA: Abscisic acid
FPKM: Fragments Per Kilobase of transcript per Million mapped reads
qRT-PCR: Quantitative real-time polymerase chain reaction

## Additional Information and Declarations

### Data Availability

The following information was supplied regarding data availability: The raw data is available in the Supplemental Files.
