# Supplementary material for: Comprehensive genome characterization and expression analysis of the DUF4228 gene family in Sorghum (Sorghum bicolor L.) under salt stress conditions
Source: PeerJ. 2026 Apr 27;14:e21175. doi: 10.7717/peerj.21175 (PMC13131349; doi:10.7717/peerj.21175)
Supplement: Supplemental Information 8 [file peerj-14-21175-s008.docx]

**Figure S1.** Protein sequence alignment of the 25 *SbDUF4228* homologs in sorghum
